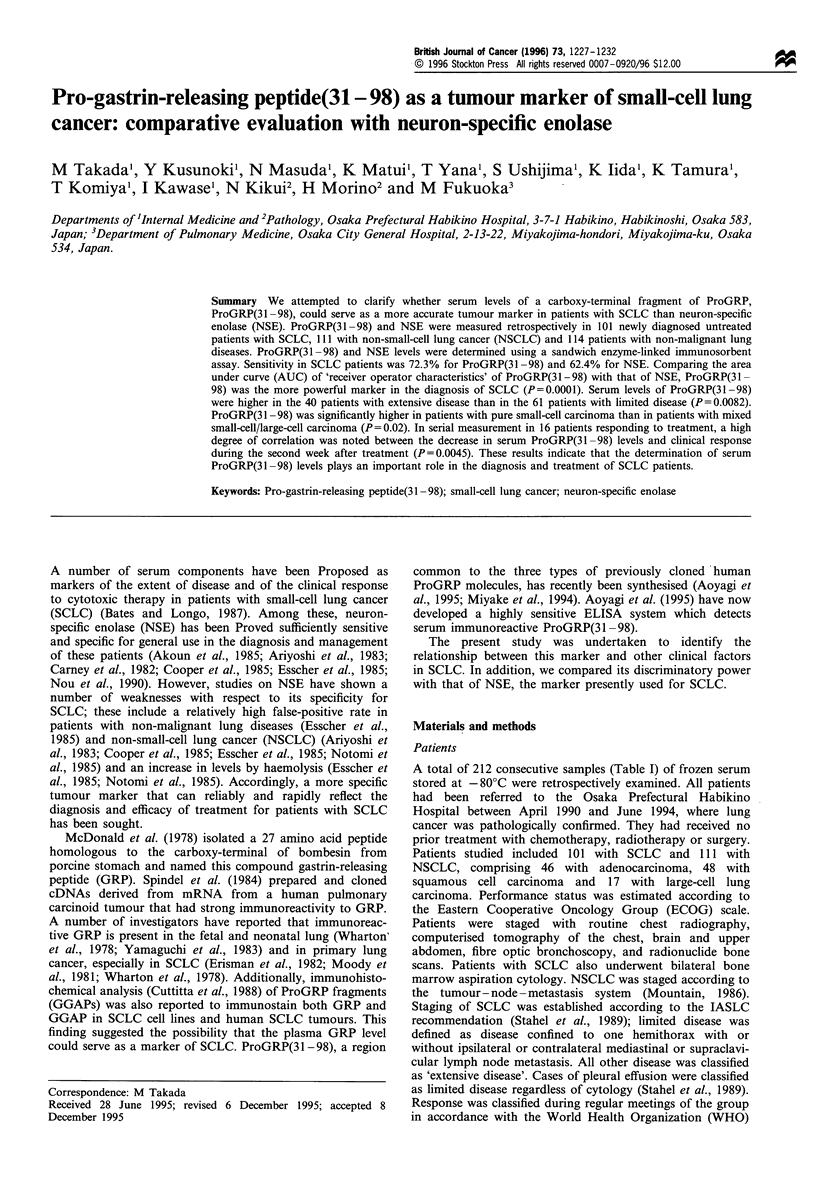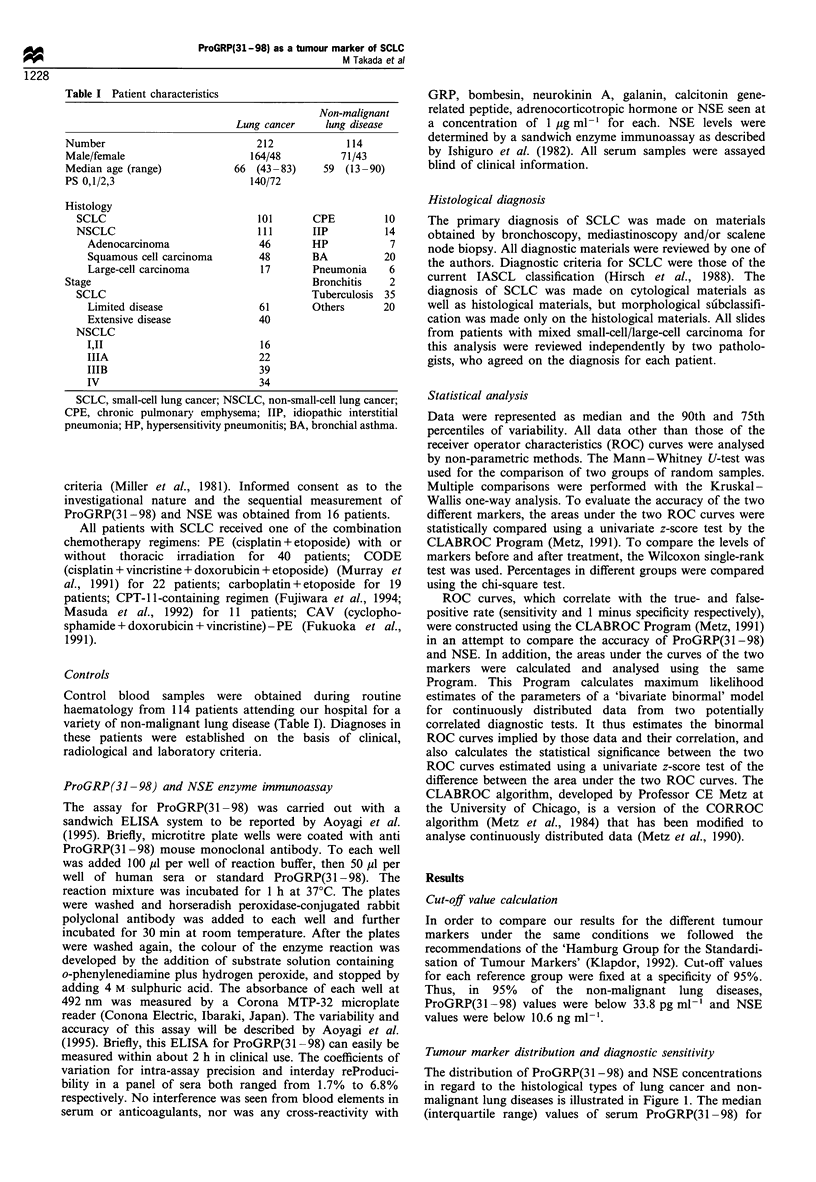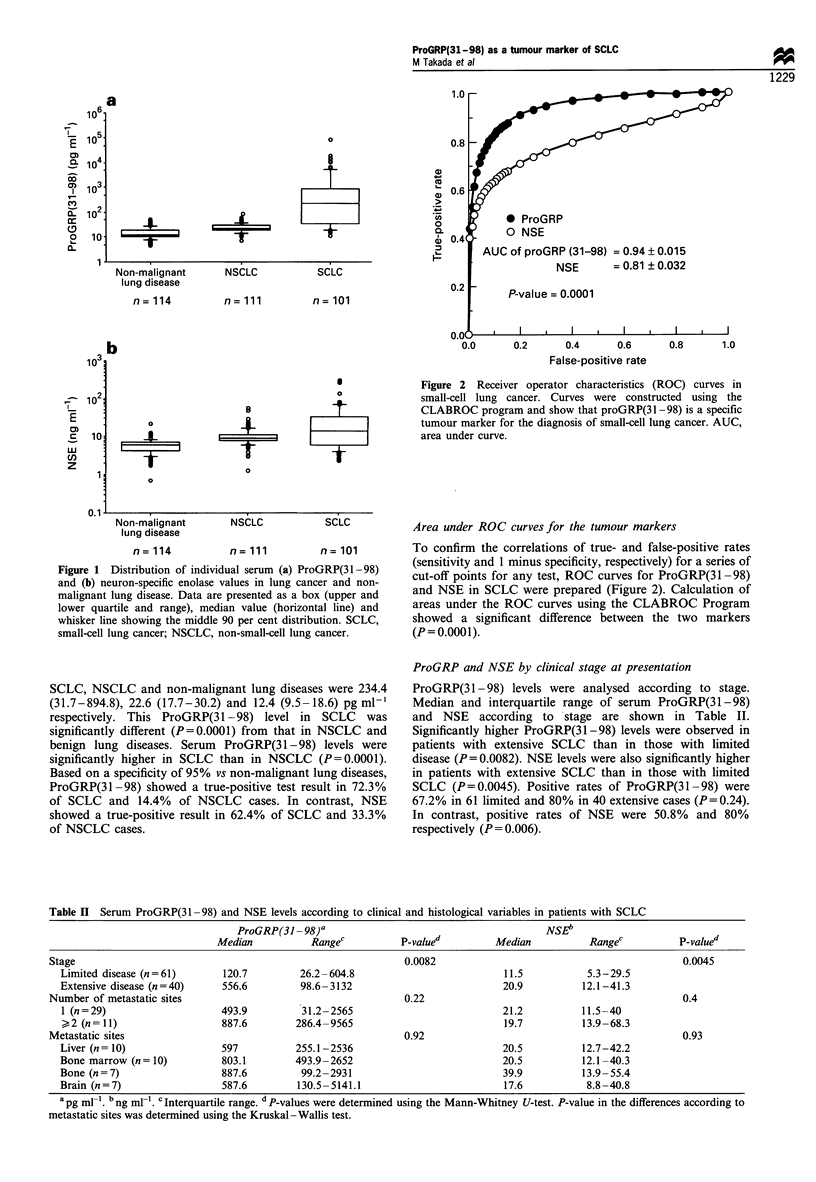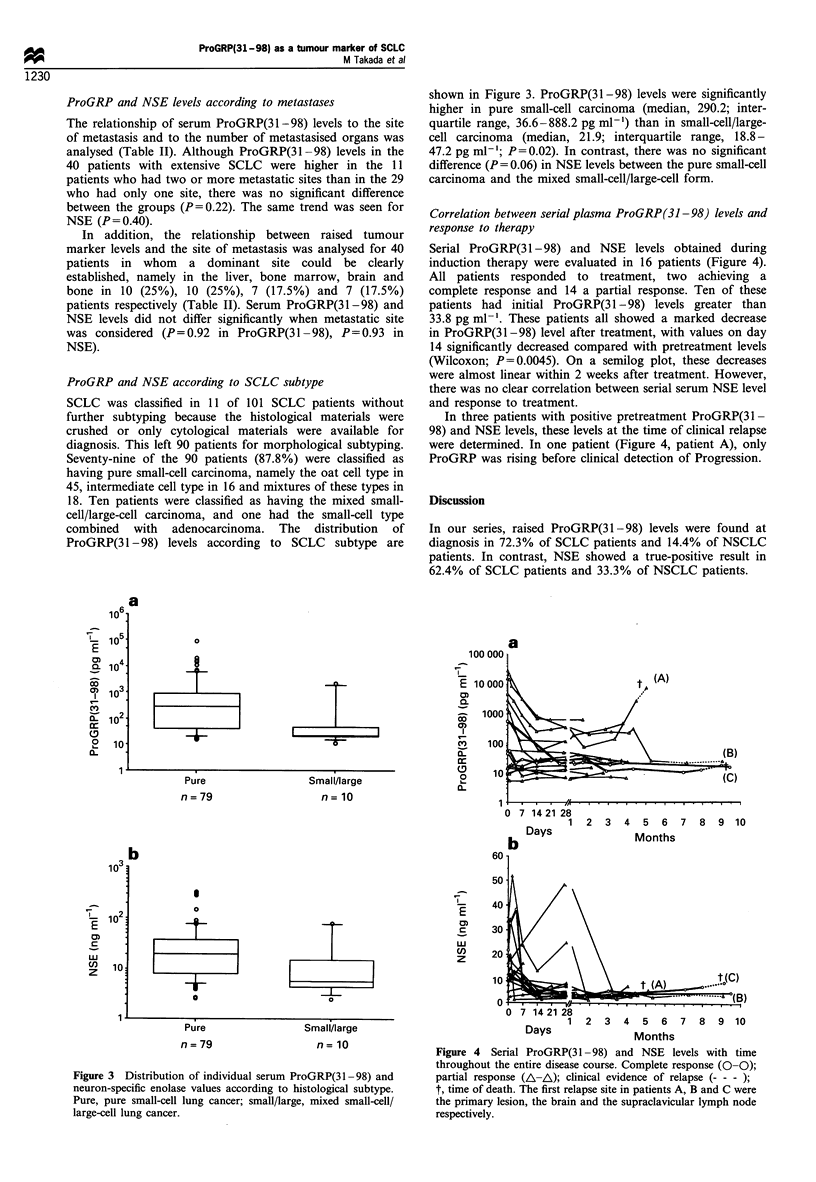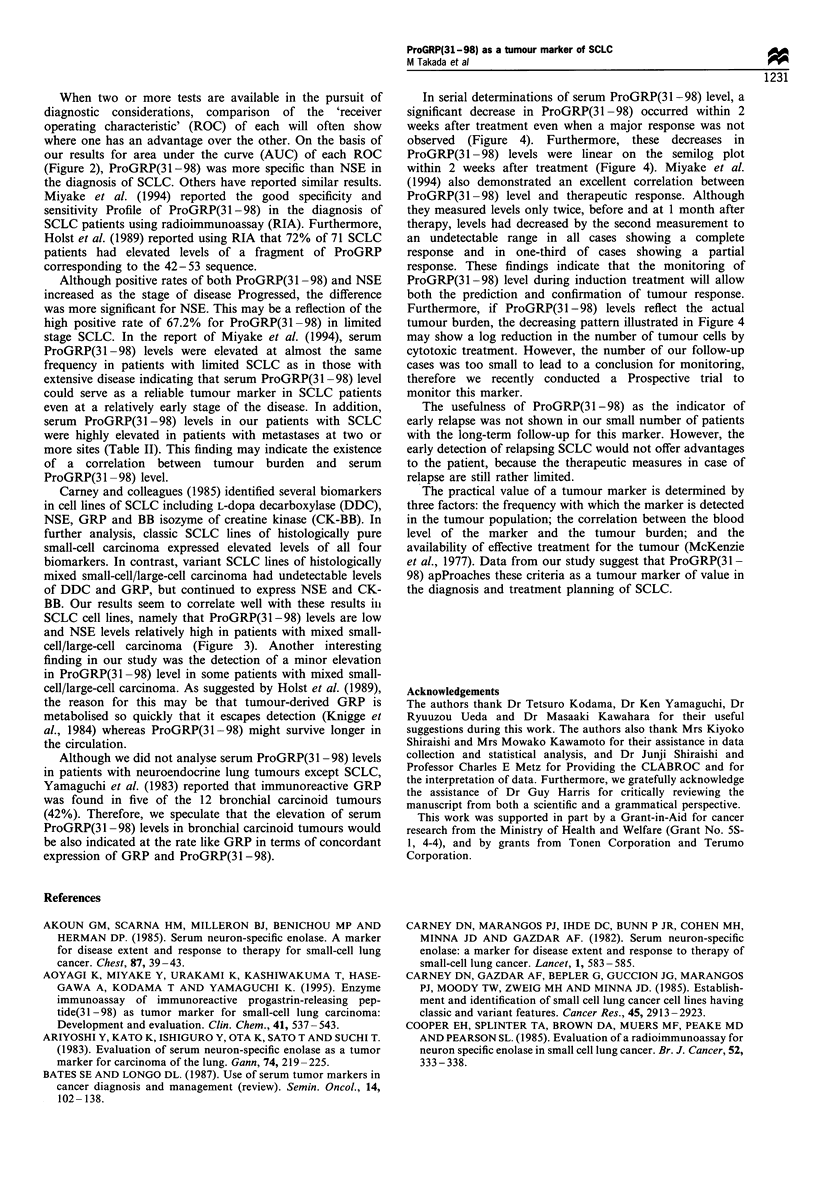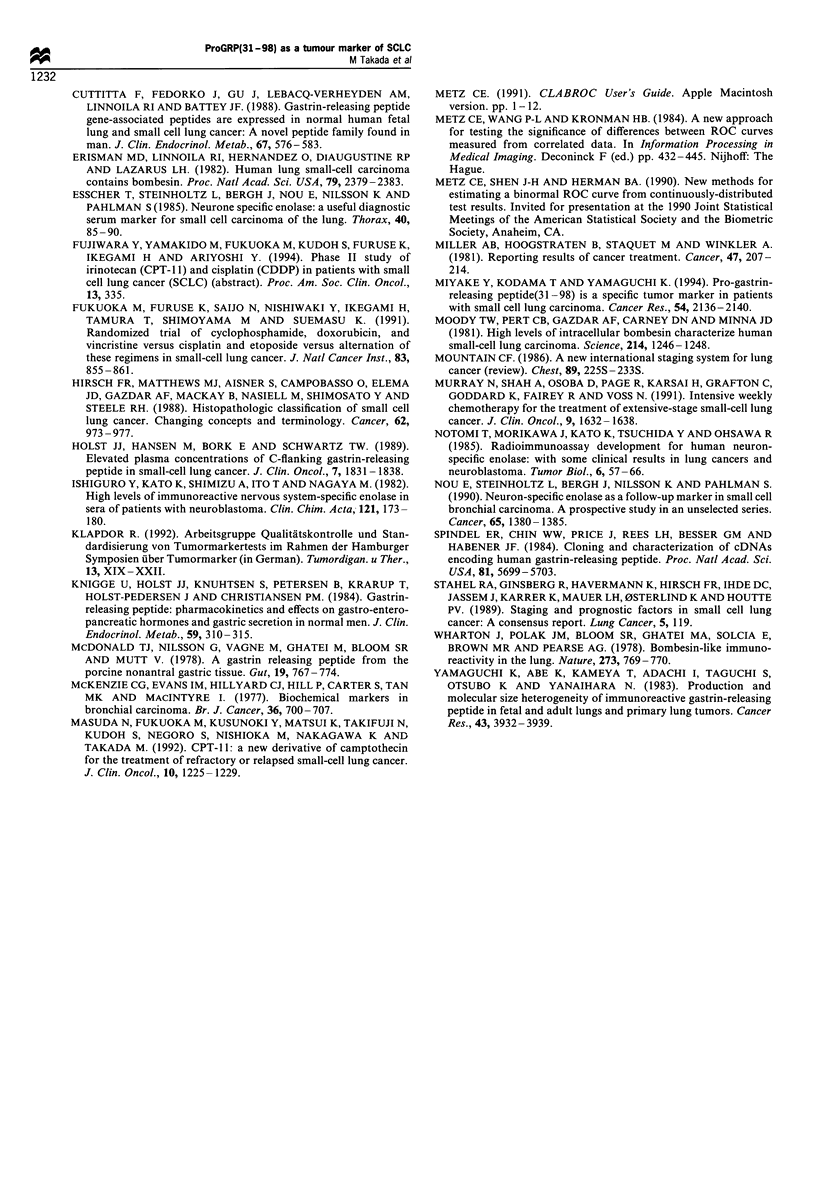# Pro-gastrin-releasing peptide (31-98) as a tumour marker of small-cell lung cancer: comparative evaluation with neuron-specific enolase.

**DOI:** 10.1038/bjc.1996.235

**Published:** 1996-05

**Authors:** M. Takada, Y. Kusunoki, N. Masuda, K. Matui, T. Yana, S. Ushijima, K. Iida, K. Tamura, T. Komiya, I. Kawase, N. Kikui, H. Morino, M. Fukuoka

**Affiliations:** Department of Internal Medicine, Osaka Prefectural Habikino Hospital, Japan.

## Abstract

We attempted to clarify whether serum levels of a carboxy-terminal fragment of ProGRP, ProGRP(31-98), could serve as a more accurate tumour marker in patients with SCLC than neuron-specific enolase (NSE). ProGRP(31-98) and NSE were measured retrospectively in 101 newly diagnosed untreated patients with SCLC, 111 with non-small-cell lung cancer (NSCLC) and 114 patients with non-malignant lung diseases. ProGRP(31-98) and NSE levels were determined using a sandwich enzyme-linked immunosorbent assay. Sensitivity in SCLC patients was 72.3% for ProGRP(31-98) and 62.4% for NSE. Comparing the area under curve (AUC) of 'receiver operator characteristics' of ProGRP(31-98) with that of NSE, ProGRP(31-98) was the more powerful marker in the diagnosis of SCLC (P = 0.0001). Serum levels of ProGRP(31-98) were higher in the 40 patients with extensive disease than in the 61 patients with limited disease (P = 0.0082). ProGRP(31-98) was significantly higher in patients with pure small-cell carcinoma than in patients with mixed small-cell/large-cell carcinoma (P = 0.02). In serial measurement in 16 patients responding to treatment, a high degree of correlation was noted between the decrease in serum ProGRP(31-98) levels and clinical response during the second week after treatment (P = 0.0045). These results indicate that the determination of serum ProGRP(31-98) levels plays an important role in the diagnosis and treatment of SCLC patients.


British Journal of Cancer (1996) 73, 1227–1232
© 1996 Stockton Press All rights reserved 0007–0920/96 $12.00

### Pro-gastrin-releasing peptide(31–98) as a tumour marker of small-cell lung cancer: comparative evaluation with neuron-specific enolase

M Takada[1], Y Kusunoki[1], N Masuda[1], K Matui[1], T Yana[1], S Ushijima[1], K Iida[1], K Tamura[1], T Komiya[1], I Kawase[1], N Kikui[2], H Morino[2] and M Fukuoka[3]

*Departments of [1]Internal Medicine and [2]Pathology, Osaka Prefectural Habikino Hospital, 3-7-1 Habikino, Habikinoshi, Osaka 583, Japan; [3]Department of Pulmonary Medicine, Osaka City General Hospital, 2-13-22, Miyakojima-hondori, Miyakojima-ku, Osaka 534, Japan.*

**Summary** We attempted to clarify whether serum levels of a carboxy-terminal fragment of ProGRP, ProGRP(31–98), could serve as a more accurate tumour marker in patients with SCLC than neuron-specific enolase (NSE). ProGRP(31–98) and NSE were measured retrospectively in 101 newly diagnosed untreated patients with SCLC, 111 with non-small-cell lung cancer (NSCLC) and 114 patients with non-malignant lung diseases. ProGRP(31–98) and NSE levels were determined using a sandwich enzyme-linked immunosorbent assay. Sensitivity in SCLC patients was 72.3% for ProGRP(31–98) and 62.4% for NSE. Comparing the area under curve (AUC) of 'receiver operator characteristics' of ProGRP(31–98) with that of NSE, ProGRP(31–98) was the more powerful marker in the diagnosis of SCLC ($P=0.0001$). Serum levels of ProGRP(31–98) were higher in the 40 patients with extensive disease than in the 61 patients with limited disease ($P=0.0082$). ProGRP(31–98) was significantly higher in patients with pure small-cell carcinoma than in patients with mixed small-cell/large-cell carcinoma ($P=0.02$). In serial measurement in 16 patients responding to treatment, a high degree of correlation was noted between the decrease in serum ProGRP(31–98) levels and clinical response during the second week after treatment ($P=0.0045$). These results indicate that the determination of serum ProGRP(31–98) levels plays an important role in the diagnosis and treatment of SCLC patients.

**Keywords:** Pro-gastrin-releasing peptide(31–98); small-cell lung cancer; neuron-specific enolase

A number of serum components have been Proposed as markers of the extent of disease and of the clinical response to cytotoxic therapy in patients with small-cell lung cancer (SCLC) (Bates and Longo, 1987). Among these, neuron-specific enolase (NSE) has been Proved sufficiently sensitive and specific for general use in the diagnosis and management of these patients (Akoun *et al.*, 1985; Ariyoshi *et al.*, 1983; Carney *et al.*, 1982; Cooper *et al.*, 1985; Esscher *et al.*, 1985; Nou *et al.*, 1990). However, studies on NSE have shown a number of weaknesses with respect to its specificity for SCLC; these include a relatively high false-positive rate in patients with non-malignant lung diseases (Esscher *et al.*, 1985) and non-small-cell lung cancer (NSCLC) (Ariyoshi *et al.*, 1983; Cooper *et al.*, 1985; Esscher *et al.*, 1985; Notomi *et al.*, 1985) and an increase in levels by haemolysis (Esscher *et al.*, 1985; Notomi *et al.*, 1985). Accordingly, a more specific tumour marker that can reliably and rapidly reflect the diagnosis and efficacy of treatment for patients with SCLC has been sought.

McDonald *et al.* (1978) isolated a 27 amino acid peptide homologous to the carboxy-terminal of bombesin from porcine stomach and named this compound gastrin-releasing peptide (GRP). Spindel *et al.* (1984) prepared and cloned cDNAs derived from mRNA from a human pulmonary carcinoid tumour that had strong immunoreactivity to GRP. A number of investigators have reported that immunoreactive GRP is present in the fetal and neonatal lung (Wharton *et al.*, 1978; Yamaguchi *et al.*, 1983) and in primary lung cancer, especially in SCLC (Erisman *et al.*, 1982; Moody *et al.*, 1981; Wharton *et al.*, 1978). Additionally, immunohistochemical analysis (Cuttitta *et al.*, 1988) of ProGRP fragments (GGAPs) was also reported to immunostain both GRP and GGAP in SCLC cell lines and human SCLC tumours. This finding suggested the possibility that the plasma GRP level could serve as a marker of SCLC. ProGRP(31–98), a region common to the three types of previously cloned human ProGRP molecules, has recently been synthesised (Aoyagi *et al.*, 1995; Miyake *et al.*, 1994). Aoyagi *et al.* (1995) have now developed a highly sensitive ELISA system which detects serum immunoreactive ProGRP(31–98).

The present study was undertaken to identify the relationship between this marker and other clinical factors in SCLC. In addition, we compared its discriminatory power with that of NSE, the marker presently used for SCLC.

### Materials and methods

### *Patients*

A total of 212 consecutive samples (Table I) of frozen serum stored at −80°C were retrospectively examined. All patients had been referred to the Osaka Prefectural Habikino Hospital between April 1990 and June 1994, where lung cancer was pathologically confirmed. They had received no prior treatment with chemotherapy, radiotherapy or surgery. Patients studied included 101 with SCLC and 111 with NSCLC, comprising 46 with adenocarcinoma, 48 with squamous cell carcinoma and 17 with large-cell lung carcinoma. Performance status was estimated according to the Eastern Cooperative Oncology Group (ECOG) scale. Patients were staged with routine chest radiography, computerised tomography of the chest, brain and upper abdomen, fibre optic bronchoscopy, and radionuclide bone scans. Patients with SCLC also underwent bilateral bone marrow aspiration cytology. NSCLC was staged according to the tumour–node–metastasis system (Mountain, 1986). Staging of SCLC was established according to the IASLC recommendation (Stahel *et al.*, 1989); limited disease was defined as disease confined to one hemithorax with or without ipsilateral or contralateral mediastinal or supraclavicular lymph node metastasis. All other disease was classified as 'extensive disease'. Cases of pleural effusion were classified as limited disease regardless of cytology (Stahel *et al.*, 1989). Response was classified during regular meetings of the group in accordance with the World Health Organization (WHO)

Correspondence: M Takada
Received 28 June 1995; revised 6 December 1995; accepted 8 December 1995

**Table I** Patient characteristics

| | Lung cancer | Non-malignant lung disease | |
|---|---|---|---|
| Number | 212 | 114 | |
| Male/female | 164/48 | 71/43 | |
| Median age (range) | 66 (43-83) | 59 (13-90) | |
| PS 0,1/2,3 | 140/72 | | |
| Histology | | | |
| SCLC | 101 | CPE | 10 |
| NSCLC | 111 | IIP | 14 |
| Adenocarcinoma | 46 | HP | 7 |
| Squamous cell carcinoma | 48 | BA | 20 |
| Large-cell carcinoma | 17 | Pneumonia | 6 |
| Stage | | Bronchitis | 2 |
| SCLC | | Tuberculosis | 35 |
| Limited disease | 61 | Others | 20 |
| Extensive disease | 40 | | |
| NSCLC | | | |
| I,II | 16 | | |
| IIIA | 22 | | |
| IIIB | 39 | | |
| IV | 34 | | |

SCLC, small-cell lung cancer; NSCLC, non-small-cell lung cancer; CPE, chronic pulmonary emphysema; IIP, idiopathic interstitial pneumonia; HP, hypersensitivity pneumonitis; BA, bronchial asthma.

criteria (Miller *et al.*, 1981). Informed consent as to the investigational nature and the sequential measurement of ProGRP(31-98) and NSE was obtained from 16 patients.

All patients with SCLC received one of the combination chemotherapy regimens: PE (cisplatin + etoposide) with or without thoracic irradiation for 40 patients; CODE (cisplatin + vincristine + doxorubicin + etoposide) (Murray *et al.*, 1991) for 22 patients; carboplatin + etoposide for 19 patients; CPT-11-containing regimen (Fujiwara *et al.*, 1994; Masuda *et al.*, 1992) for 11 patients; CAV (cyclophosphamide + doxorubicin + vincristine)-PE (Fukuoka *et al.*, 1991).

*Controls*

Control blood samples were obtained during routine haematology from 114 patients attending our hospital for a variety of non-malignant lung disease (Table I). Diagnoses in these patients were established on the basis of clinical, radiological and laboratory criteria.

*ProGRP(31-98) and NSE enzyme immunoassay*

The assay for ProGRP(31-98) was carried out with a sandwich ELISA system to be reported by Aoyagi *et al.* (1995). Briefly, microtitre plate wells were coated with anti ProGRP(31-98) mouse monoclonal antibody. To each well was added 100 $\mu$l per well of reaction buffer, then 50 $\mu$l per well of human sera or standard ProGRP(31-98). The reaction mixture was incubated for 1 h at 37°C. The plates were washed and horseradish peroxidase-conjugated rabbit polyclonal antibody was added to each well and further incubated for 30 min at room temperature. After the plates were washed again, the colour of the enzyme reaction was developed by the addition of substrate solution containing *o*-phenylenediamine plus hydrogen peroxide, and stopped by adding 4 M sulphuric acid. The absorbance of each well at 492 nm was measured by a Corona MTP-32 microplate reader (Conona Electric, Ibaraki, Japan). The variability and accuracy of this assay will be described by Aoyagi *et al.* (1995). Briefly, this ELISA for ProGRP(31-98) can easily be measured within about 2 h in clinical use. The coefficients of variation for intra-assay precision and interday reProducibility in a panel of sera both ranged from 1.7% to 6.8% respectively. No interference was seen from blood elements in serum or anticoagulants, nor was any cross-reactivity with GRP, bombesin, neurokinin A, galanin, calcitonin gene-related peptide, adrenocorticotropic hormone or NSE seen at a concentration of 1 $\mu$g ml$^{-1}$ for each. NSE levels were determined by a sandwich enzyme immunoassay as described by Ishiguro *et al.* (1982). All serum samples were assayed blind of clinical information.

*Histological diagnosis*

The primary diagnosis of SCLC was made on materials obtained by bronchoscopy, mediastinoscopy and/or scalene node biopsy. All diagnostic materials were reviewed by one of the authors. Diagnostic criteria for SCLC were those of the current IASCL classification (Hirsch *et al.*, 1988). The diagnosis of SCLC was made on cytological materials as well as histological materials, but morphological sübclassification was made only on the histological materials. All slides from patients with mixed small-cell/large-cell carcinoma for this analysis were reviewed independently by two pathologists, who agreed on the diagnosis for each patient.

*Statistical analysis*

Data were represented as median and the 90th and 75th percentiles of variability. All data other than those of the receiver operator characteristics (ROC) curves were analysed by non-parametric methods. The Mann-Whitney *U*-test was used for the comparison of two groups of random samples. Multiple comparisons were performed with the Kruskal-Wallis one-way analysis. To evaluate the accuracy of the two different markers, the areas under the two ROC curves were statistically compared using a univariate *z*-score test by the CLABROC Program (Metz, 1991). To compare the levels of markers before and after treatment, the Wilcoxon single-rank test was used. Percentages in different groups were compared using the chi-square test.

ROC curves, which correlate with the true- and false-positive rate (sensitivity and 1 minus specificity respectively), were constructed using the CLABROC Program (Metz, 1991) in an attempt to compare the accuracy of ProGRP(31-98) and NSE. In addition, the areas under the curves of the two markers were calculated and analysed using the same Program. This Program calculates maximum likelihood estimates of the parameters of a 'bivariate binormal' model for continuously distributed data from two potentially correlated diagnostic tests. It thus estimates the binormal ROC curves implied by those data and their correlation, and also calculates the statistical significance between the two ROC curves estimated using a univariate *z*-score test of the difference between the area under the two ROC curves. The CLABROC algorithm, developed by Professor CE Metz at the University of Chicago, is a version of the CORROC algorithm (Metz *et al.*, 1984) that has been modified to analyse continuously distributed data (Metz *et al.*, 1990).

### Results

*Cut-off value calculation*

In order to compare our results for the different tumour markers under the same conditions we followed the recommendations of the 'Hamburg Group for the Standardisation of Tumour Markers' (Klapdor, 1992). Cut-off values for each reference group were fixed at a specificity of 95%. Thus, in 95% of the non-malignant lung diseases, ProGRP(31-98) values were below 33.8 pg ml$^{-1}$ and NSE values were below 10.6 ng ml$^{-1}$.

*Tumour marker distribution and diagnostic sensitivity*

The distribution of ProGRP(31-98) and NSE concentrations in regard to the histological types of lung cancer and non-malignant lung diseases is illustrated in Figure 1. The median (interquartile range) values of serum ProGRP(31-98) for

**Figure 1** Distribution of individual serum (**a**) ProGRP(31–98) and (**b**) neuron-specific enolase values in lung cancer and non-malignant lung disease. Data are presented as a box (upper and lower quartile and range), median value (horizontal line) and whisker line showing the middle 90 per cent distribution. SCLC, small-cell lung cancer; NSCLC, non-small-cell lung cancer.

**Figure 2** Receiver operator characteristics (ROC) curves in small-cell lung cancer. Curves were constructed using the CLABROC program and show that proGRP(31–98) is a specific tumour marker for the diagnosis of small-cell lung cancer. AUC, area under curve.

SCLC, NSCLC and non-malignant lung diseases were 234.4 (31.7–894.8), 22.6 (17.7–30.2) and 12.4 (9.5–18.6) pg ml$^{-1}$ respectively. This ProGRP(31–98) level in SCLC was significantly different ($P=0.0001$) from that in NSCLC and benign lung diseases. Serum ProGRP(31–98) levels were significantly higher in SCLC than in NSCLC ($P=0.0001$). Based on a specificity of 95% *vs* non-malignant lung diseases, ProGRP(31–98) showed a true-positive test result in 72.3% of SCLC and 14.4% of NSCLC cases. In contrast, NSE showed a true-positive result in 62.4% of SCLC and 33.3% of NSCLC cases.

### *Area under ROC curves for the tumour markers*

To confirm the correlations of true- and false-positive rates (sensitivity and 1 minus specificity, respectively) for a series of cut-off points for any test, ROC curves for ProGRP(31–98) and NSE in SCLC were prepared (Figure 2). Calculation of areas under the ROC curves using the CLABROC Program showed a significant difference between the two markers ($P=0.0001$).

### *ProGRP and NSE by clinical stage at presentation*

ProGRP(31–98) levels were analysed according to stage. Median and interquartile range of serum ProGRP(31–98) and NSE according to stage are shown in Table II. Significantly higher ProGRP(31–98) levels were observed in patients with extensive SCLC than in those with limited disease ($P=0.0082$). NSE levels were also significantly higher in patients with extensive SCLC than in those with limited SCLC ($P=0.0045$). Positive rates of ProGRP(31–98) were 67.2% in 61 limited and 80% in 40 extensive cases ($P=0.24$). In contrast, positive rates of NSE were 50.8% and 80% respectively ($P=0.006$).

**Table II** Serum ProGRP(31–98) and NSE levels according to clinical and histological variables in patients with SCLC

| | *ProGRP(31–98)*[a] | | | *NSE*[b] | | |
|---|---|---|---|---|---|---|
| | *Median* | *Range*[c] | P-*value*[d] | *Median* | *Range*[c] | P-*value*[d] |
| Stage | | | 0.0082 | | | 0.0045 |
| Limited disease ($n=61$) | 120.7 | 26.2–604.8 | | 11.5 | 5.3–29.5 | |
| Extensive disease ($n=40$) | 556.6 | 98.6–3132 | | 20.9 | 12.1–41.3 | |
| Number of metastatic sites | | | 0.22 | | | 0.4 |
| 1 ($n=29$) | 493.9 | 31.2–2565 | | 21.2 | 11.5–40 | |
| ⩾2 ($n=11$) | 887.6 | 286.4–9565 | | 19.7 | 13.9–68.3 | |
| Metastatic sites | | | 0.92 | | | 0.93 |
| Liver ($n=10$) | 597 | 255.1–2536 | | 20.5 | 12.7–42.2 | |
| Bone marrow ($n=10$) | 803.1 | 493.9–2652 | | 20.5 | 12.1–40.3 | |
| Bone ($n=7$) | 887.6 | 99.2–2931 | | 39.9 | 13.9–55.4 | |
| Brain ($n=7$) | 587.6 | 130.5–5141.1 | | 17.6 | 8.8–40.8 | |

[a]pg ml$^{-1}$. [b]ng ml$^{-1}$. [c]Interquartile range. [d]*P*-values were determined using the Mann-Whitney *U*-test. *P*-value in the differences according to metastatic sites was determined using the Kruskal–Wallis test.

*ProGRP and NSE levels according to metastases*

The relationship of serum ProGRP(31-98) levels to the site of metastasis and to the number of metastasised organs was analysed (Table II). Although ProGRP(31-98) levels in the 40 patients with extensive SCLC were higher in the 11 patients who had two or more metastatic sites than in the 29 who had only one site, there was no significant difference between the groups ($P=0.22$). The same trend was seen for NSE ($P=0.40$).

In addition, the relationship between raised tumour marker levels and the site of metastasis was analysed for 40 patients in whom a dominant site could be clearly established, namely in the liver, bone marrow, brain and bone in 10 (25%), 10 (25%), 7 (17.5%) and 7 (17.5%) patients respectively (Table II). Serum ProGRP(31-98) and NSE levels did not differ significantly when metastatic site was considered ($P=0.92$ in ProGRP(31-98), $P=0.93$ in NSE).

*ProGRP and NSE according to SCLC subtype*

SCLC was classified in 11 of 101 SCLC patients without further subtyping because the histological materials were crushed or only cytological materials were available for diagnosis. This left 90 patients for morphological subtyping. Seventy-nine of the 90 patients (87.8%) were classified as having pure small-cell carcinoma, namely the oat cell type in 45, intermediate cell type in 16 and mixtures of these types in 18. Ten patients were classified as having the mixed small-cell/large-cell carcinoma, and one had the small-cell type combined with adenocarcinoma. The distribution of ProGRP(31-98) levels according to SCLC subtype are shown in Figure 3. ProGRP(31-98) levels were significantly higher in pure small-cell carcinoma (median, 290.2; interquartile range, 36.6-888.2 pg $ml^{-1}$) than in small-cell/large-cell carcinoma (median, 21.9; interquartile range, 18.8-47.2 pg $ml^{-1}$; $P=0.02$). In contrast, there was no significant difference ($P=0.06$) in NSE levels between the pure small-cell carcinoma and the mixed small-cell/large-cell form.

*Correlation between serial plasma ProGRP(31-98) levels and response to therapy*

Serial ProGRP(31-98) and NSE levels obtained during induction therapy were evaluated in 16 patients (Figure 4). All patients responded to treatment, two achieving a complete response and 14 a partial response. Ten of these patients had initial ProGRP(31-98) levels greater than 33.8 pg $ml^{-1}$. These patients all showed a marked decrease in ProGRP(31-98) level after treatment, with values on day 14 significantly decreased compared with pretreatment levels (Wilcoxon; $P=0.0045$). On a semilog plot, these decreases were almost linear within 2 weeks after treatment. However, there was no clear correlation between serial serum NSE level and response to treatment.

In three patients with positive pretreatment ProGRP(31-98) and NSE levels, these levels at the time of clinical relapse were determined. In one patient (Figure 4, patient A), only ProGRP was rising before clinical detection of Progression.

### Discussion

In our series, raised ProGRP(31-98) levels were found at diagnosis in 72.3% of SCLC patients and 14.4% of NSCLC patients. In contrast, NSE showed a true-positive result in 62.4% of SCLC patients and 33.3% of NSCLC patients.

**Figure 3** Distribution of individual serum ProGRP(31-98) and neuron-specific enolase values according to histological subtype. Pure, pure small-cell lung cancer; small/large, mixed small-cell/large-cell lung cancer.

**Figure 4** Serial ProGRP(31-98) and NSE levels with time throughout the entire disease course. Complete response (○-○); partial response (△-△); clinical evidence of relapse (- - - ); †, time of death. The first relapse site in patients A, B and C were the primary lesion, the brain and the supraclavicular lymph node respectively.

When two or more tests are available in the pursuit of diagnostic considerations, comparison of the 'receiver operating characteristic' (ROC) of each will often show where one has an advantage over the other. On the basis of our results for area under the curve (AUC) of each ROC (Figure 2), ProGRP(31-98) was more specific than NSE in the diagnosis of SCLC. Others have reported similar results. Miyake *et al.* (1994) reported the good specificity and sensitivity Profile of ProGRP(31-98) in the diagnosis of SCLC patients using radioimmunoassay (RIA). Furthermore, Holst *et al.* (1989) reported using RIA that 72% of 71 SCLC patients had elevated levels of a fragment of ProGRP corresponding to the 42-53 sequence.

Although positive rates of both ProGRP(31-98) and NSE increased as the stage of disease Progressed, the difference was more significant for NSE. This may be a reflection of the high positive rate of 67.2% for ProGRP(31-98) in limited stage SCLC. In the report of Miyake *et al.* (1994), serum ProGRP(31-98) levels were elevated at almost the same frequency in patients with limited SCLC as in those with extensive disease indicating that serum ProGRP(31-98) level could serve as a reliable tumour marker in SCLC patients even at a relatively early stage of the disease. In addition, serum ProGRP(31-98) levels in our patients with SCLC were highly elevated in patients with metastases at two or more sites (Table II). This finding may indicate the existence of a correlation between tumour burden and serum ProGRP(31-98) level.

Carney and colleagues (1985) identified several biomarkers in cell lines of SCLC including L-dopa decarboxylase (DDC), NSE, GRP and BB isozyme of creatine kinase (CK-BB). In further analysis, classic SCLC lines of histologically pure small-cell carcinoma expressed elevated levels of all four biomarkers. In contrast, variant SCLC lines of histologically mixed small-cell/large-cell carcinoma had undetectable levels of DDC and GRP, but continued to express NSE and CK-BB. Our results seem to correlate well with these results in SCLC cell lines, namely that ProGRP(31-98) levels are low and NSE levels relatively high in patients with mixed small-cell/large-cell carcinoma (Figure 3). Another interesting finding in our study was the detection of a minor elevation in ProGRP(31-98) level in some patients with mixed small-cell/large-cell carcinoma. As suggested by Holst *et al.* (1989), the reason for this may be that tumour-derived GRP is metabolised so quickly that it escapes detection (Knigge *et al.*, 1984) whereas ProGRP(31-98) might survive longer in the circulation.

Although we did not analyse serum ProGRP(31-98) levels in patients with neuroendocrine lung tumours except SCLC, Yamaguchi *et al.* (1983) reported that immunoreactive GRP was found in five of the 12 bronchial carcinoid tumours (42%). Therefore, we speculate that the elevation of serum ProGRP(31-98) levels in bronchial carcinoid tumours would be also indicated at the rate like GRP in terms of concordant expression of GRP and ProGRP(31-98).

In serial determinations of serum ProGRP(31-98) level, a significant decrease in ProGRP(31-98) occurred within 2 weeks after treatment even when a major response was not observed (Figure 4). Furthermore, these decreases in ProGRP(31-98) levels were linear on the semilog plot within 2 weeks after treatment (Figure 4). Miyake *et al.* (1994) also demonstrated an excellent correlation between ProGRP(31-98) level and therapeutic response. Although they measured levels only twice, before and at 1 month after therapy, levels had decreased by the second measurement to an undetectable range in all cases showing a complete response and in one-third of cases showing a partial response. These findings indicate that the monitoring of ProGRP(31-98) level during induction treatment will allow both the prediction and confirmation of tumour response. Furthermore, if ProGRP(31-98) levels reflect the actual tumour burden, the decreasing pattern illustrated in Figure 4 may show a log reduction in the number of tumour cells by cytotoxic treatment. However, the number of our follow-up cases was too small to lead to a conclusion for monitoring, therefore we recently conducted a Prospective trial to monitor this marker.

The usefulness of ProGRP(31-98) as the indicator of early relapse was not shown in our small number of patients with the long-term follow-up for this marker. However, the early detection of relapsing SCLC would not offer advantages to the patient, because the therapeutic measures in case of relapse are still rather limited.

The practical value of a tumour marker is determined by three factors: the frequency with which the marker is detected in the tumour population; the correlation between the blood level of the marker and the tumour burden; and the availability of effective treatment for the tumour (McKenzie *et al.*, 1977). Data from our study suggest that ProGRP(31-98) apProaches these criteria as a tumour marker of value in the diagnosis and treatment planning of SCLC.

**Acknowledgements**

The authors thank Dr Tetsuro Kodama, Dr Ken Yamaguchi, Dr Ryuuzou Ueda and Dr Masaaki Kawahara for their useful suggestions during this work. The authors also thank Mrs Kiyoko Shiraishi and Mrs Mowako Kawamoto for their assistance in data collection and statistical analysis, and Dr Junji Shiraishi and Professor Charles E Metz for Providing the CLABROC and for the interpretation of data. Furthermore, we gratefully acknowledge the assistance of Dr Guy Harris for critically reviewing the manuscript from both a scientific and a grammatical perspective.

This work was supported in part by a Grant-in-Aid for cancer research from the Ministry of Health and Welfare (Grant No. 5S-1, 4-4), and by grants from Tonen Corporation and Terumo Corporation.

### References

AKOUN GM, SCARNA HM, MILLERON BJ, BENICHOU MP AND HERMAN DP. (1985). Serum neuron-specific enolase. A marker for disease extent and response to therapy for small-cell lung cancer. *Chest*, **87**, 39-43.

AOYAGI K, MIYAKE Y, URAKAMI K, KASHIWAKUMA T, HASEGAWA A, KODAMA T AND YAMAGUCHI K. (1995). Enzyme immunoassay of immunoreactive progastrin-releasing peptide(31-98) as tumor marker for small-cell lung carcinoma: Development and evaluation. *Clin. Chem.*, **41**, 537-543.

ARIYOSHI Y, KATO K, ISHIGURO Y, OTA K, SATO T AND SUCHI T. (1983). Evaluation of serum neuron-specific enolase as a tumor marker for carcinoma of the lung. *Gann*, **74**, 219-225.

BATES SE AND LONGO DL. (1987). Use of serum tumor markers in cancer diagnosis and management (review). *Semin. Oncol.*, **14**, 102-138.

CARNEY DN, MARANGOS PJ, IHDE DC, BUNN P JR, COHEN MH, MINNA JD AND GAZDAR AF. (1982). Serum neuron-specific enolase: a marker for disease extent and response to therapy of small-cell lung cancer. *Lancet*, **1**, 583-585.

CARNEY DN, GAZDAR AF, BEPLER G, GUCCION JG, MARANGOS PJ, MOODY TW, ZWEIG MH AND MINNA JD. (1985). Establishment and identification of small cell lung cancer cell lines having classic and variant features. *Cancer Res.*, **45**, 2913-2923.

COOPER EH, SPLINTER TA, BROWN DA, MUERS MF, PEAKE MD AND PEARSON SL. (1985). Evaluation of a radioimmunoassay for neuron specific enolase in small cell lung cancer. *Br. J. Cancer*, **52**, 333-338.

CUTTITTA F, FEDORKO J, GU J, LEBACQ-VERHEYDEN AM, LINNOILA RI AND BATTEY JF. (1988). Gastrin-releasing peptide gene-associated peptides are expressed in normal human fetal lung and small cell lung cancer: A novel peptide family found in man. *J. Clin. Endocrinol. Metab.*, **67**, 576–583.

ERISMAN MD, LINNOILA RI, HERNANDEZ O, DIAUGUSTINE RP AND LAZARUS LH. (1982). Human lung small-cell carcinoma contains bombesin. *Proc. Natl Acad. Sci. USA*, **79**, 2379–2383.

ESSCHER T, STEINHOLTZ L, BERGH J, NOU E, NILSSON K AND PAHLMAN S (1985). Neurone specific enolase: a useful diagnostic serum marker for small cell carcinoma of the lung. *Thorax*, **40**, 85–90.

FUJIWARA Y, YAMAKIDO M, FUKUOKA M, KUDOH S, FURUSE K, IKEGAMI H AND ARIYOSHI Y. (1994). Phase II study of irinotecan (CPT-11) and cisplatin (CDDP) in patients with small cell lung cancer (SCLC) (abstract). *Proc. Am. Soc. Clin. Oncol.*, **13**, 335.

FUKUOKA M, FURUSE K, SAIJO N, NISHIWAKI Y, IKEGAMI H, TAMURA T, SHIMOYAMA M AND SUEMASU K. (1991). Randomized trial of cyclophosphamide, doxorubicin, and vincristine versus cisplatin and etoposide versus alternation of these regimens in small-cell lung cancer. *J. Natl Cancer Inst.*, **83**, 855–861.

HIRSCH FR, MATTHEWS MJ, AISNER S, CAMPOBASSO O, ELEMA JD, GAZDAR AF, MACKAY B, NASIELL M, SHIMOSATO Y AND STEELE RH. (1988). Histopathologic classification of small cell lung cancer. Changing concepts and terminology. *Cancer*, **62**, 973–977.

HOLST JJ, HANSEN M, BORK E AND SCHWARTZ TW. (1989). Elevated plasma concentrations of C-flanking gastrin-releasing peptide in small-cell lung cancer. *J. Clin. Oncol.*, **7**, 1831–1838.

ISHIGURO Y, KATO K, SHIMIZU A, ITO T AND NAGAYA M. (1982). High levels of immunoreactive nervous system-specific enolase in sera of patients with neuroblastoma. *Clin. Chim. Acta*, **121**, 173–180.

KLAPDOR R. (1992). Arbeitsgruppe Qualitätskontrolle und Standardisierung von Tumormarkertests im Rahmen der Hamburger Symposien über Tumormarker (in German). *Tumordigan. u Ther.*, **13**, XIX–XXII.

KNIGGE U, HOLST JJ, KNUHTSEN S, PETERSEN B, KRARUP T, HOLST-PEDERSEN J AND CHRISTIANSEN PM. (1984). Gastrin-releasing peptide: pharmacokinetics and effects on gastro-entero-pancreatic hormones and gastric secretion in normal men. *J. Clin. Endocrinol. Metab.*, **59**, 310–315.

McDONALD TJ, NILSSON G, VAGNE M, GHATEI M, BLOOM SR AND MUTT V. (1978). A gastrin releasing peptide from the porcine nonantral gastric tissue. *Gut*, **19**, 767–774.

McKENZIE CG, EVANS IM, HILLYARD CJ, HILL P, CARTER S, TAN MK AND MacINTYRE I. (1977). Biochemical markers in bronchial carcinoma. *Br. J. Cancer*, **36**, 700–707.

MASUDA N, FUKUOKA M, KUSUNOKI Y, MATSUI K, TAKIFUJI N, KUDOH S, NEGORO S, NISHIOKA M, NAKAGAWA K AND TAKADA M. (1992). CPT-11: a new derivative of camptothecin for the treatment of refractory or relapsed small-cell lung cancer. *J. Clin. Oncol.*, **10**, 1225–1229.

METZ CE. (1991). *CLABROC User's Guide*. Apple Macintosh version. pp. 1–12.

METZ CE, WANG P-L AND KRONMAN HB. (1984). A new approach for testing the significance of differences between ROC curves measured from correlated data. In *Information Processing in Medical Imaging*. Deconinck F (ed.) pp. 432–445. Nijhoff: The Hague.

METZ CE, SHEN J-H AND HERMAN BA. (1990). New methods for estimating a binormal ROC curve from continuously-distributed test results. Invited for presentation at the 1990 Joint Statistical Meetings of the American Statistical Society and the Biometric Society, Anaheim, CA.

MILLER AB, HOOGSTRATEN B, STAQUET M AND WINKLER A. (1981). Reporting results of cancer treatment. *Cancer*, **47**, 207–214.

MIYAKE Y, KODAMA T AND YAMAGUCHI K. (1994). Pro-gastrin-releasing peptide(31–98) is a specific tumor marker in patients with small cell lung carcinoma. *Cancer Res.*, **54**, 2136–2140.

MOODY TW, PERT CB, GAZDAR AF, CARNEY DN AND MINNA JD (1981). High levels of intracellular bombesin characterize human small-cell lung carcinoma. *Science*, **214**, 1246–1248.

MOUNTAIN CF. (1986). A new international staging system for lung cancer (review). *Chest*, **89**, 225S–233S.

MURRAY N, SHAH A, OSOBA D, PAGE R, KARSAI H, GRAFTON C, GODDARD K, FAIREY R AND VOSS N. (1991). Intensive weekly chemotherapy for the treatment of extensive-stage small-cell lung cancer. *J. Clin. Oncol.*, **9**, 1632–1638.

NOTOMI T, MORIKAWA J, KATO K, TSUCHIDA Y AND OHSAWA R (1985). Radioimmunoassay development for human neuron-specific enolase: with some clinical results in lung cancers and neuroblastoma. *Tumor Biol.*, **6**, 57–66.

NOU E, STEINHOLTZ L, BERGH J, NILSSON K AND PAHLMAN S. (1990). Neuron-specific enolase as a follow-up marker in small cell bronchial carcinoma. A prospective study in an unselected series. *Cancer*, **65**, 1380–1385.

SPINDEL ER, CHIN WW, PRICE J, REES LH, BESSER GM AND HABENER JF. (1984). Cloning and characterization of cDNAs encoding human gastrin-releasing peptide. *Proc. Natl Acad. Sci. USA*, **81**, 5699–5703.

STAHEL RA, GINSBERG R, HAVERMANN K, HIRSCH FR, IHDE DC, JASSEM J, KARRER K, MAUER LH, ØSTERLIND K AND HOUTTE PV. (1989). Staging and prognostic factors in small cell lung cancer: A consensus report. *Lung Cancer*, **5**, 119.

WHARTON J, POLAK JM, BLOOM SR, GHATEI MA, SOLCIA E, BROWN MR AND PEARSE AG. (1978). Bombesin-like immunoreactivity in the lung. *Nature*, **273**, 769–770.

YAMAGUCHI K, ABE K, KAMEYA T, ADACHI I, TAGUCHI S, OTSUBO K AND YANAIHARA N. (1983). Production and molecular size heterogeneity of immunoreactive gastrin-releasing peptide in fetal and adult lungs and primary lung tumors. *Cancer Res.*, **43**, 3932–3939.